# Evaluation of the Analytical Performances of the Biolabo SOLEA 100 Optical Coagulometer and Comparison with the Stago STA-R MAX Analyser in the Determination of PT, APTT, and Fibrinogen

**DOI:** 10.3390/diagnostics13010085

**Published:** 2022-12-28

**Authors:** Pierangelo Bellio, Simonetta De Angelis, Alessandra Piccirilli, Giulio Di Michele, Remo Barnabei, Gianfranco Amicosante, Mariagrazia Perilli, Giuseppe Celenza

**Affiliations:** 1 Department of Biotechnological and Applied Clinical Sciences, University of L’Aquila, 67100 L’Aquila, Italy; 2 Clinical Laboratory, Regional Hospital “San Salvatore”, 67100 L’Aquila, Italy

**Keywords:** analytical techniques and equipment, haematology and coagulation, mechanical clot detection, optical clot detection, validation/evaluation

## Abstract

Introduction. The Biolabo Solea 100 is a fully automated coagulation analyser using an optical system to detect coagulation designed to meet the needs of small- and medium-sized laboratories. This study aimed to evaluate the analytical performance in terms of bias, precision, and interference of the Biolabo Solea 100 coagulometer under routine laboratory conditions. In addition, a comparison was made with Stago STA-R MAX. Materials and Methods. Imprecision and bias were evaluated for activated partial thromboplastin time (APTT), fibrinogen (FIB), and prothrombin time (PT) at the medical decision levels. The results of 200, 181, and 206 plasma samples for APTT, FIB, and PT, respectively, were compared with those obtained by Stago STA-R MAX. In addition, the interference level of bilirubin, haemoglobin, triglycerides, and fractionated heparin was evaluated. Results. Repeatability, intermediate imprecision, bias, and total error are overall below the defined limits of acceptability. Of interest is the high degree of agreement between Solea 100 and STA-R MAX with respect to PT (s), which fits perfectly with the theoretical line of identity (y = 0 + 1.00x). No interferences were found within the limits stated by the manufacturer, with some exceptions for APTT with heparin and APTT and PT for higher bilirubin concentrations. Conclusions. In conclusion, the performance of the Solea 100 optical analyser is satisfactory and adequate for the determination of routine coagulation tests. Moreover, they are perfectly comparable to mechanical systems, such as STA-R MAX and other upper-level analysers, even considering the low interference levels under routine conditions.

## 1. Introduction

Quality in laboratory medicine rests on two closely related aspects: efficiency and effectiveness. The first is based on the analytical accuracy and reliability of the results, the ability to deliver and communicate results promptly, and, finally, cost containment. The second is based on three main components: adequate diagnostic accuracy, clinical utility, and patient benefit, thus ensuring effective patient care [1].

Efficiency relies on the ability of the clinical laboratory to control the quality of the entire analytical process strictly. In this context, laboratory specialists must know the performance characteristics of the method they decide to use. It is not by chance that the international standard ISO15189:2012 “Medical laboratories—Requirements for quality and competence” demands that laboratories evaluate each novel assay and instrument before it can be introduced in clinical practice [2]. Paragraph 5.5.1.2, “Verification of examination procedures”, states that “validated examination procedures used without modification shall be subject to independent verification by the laboratory before being introduced into routine use”. Moreover, “The independent verification by the laboratory shall confirm, through obtaining objective evidence (in the form of performance characteristics) that the performance claims for the examination procedure have been met”.

The evaluation of the analytical performance of a method or instrument is a good laboratory practice, which becomes stringent for the accreditation of a laboratory test under the ISO15189:2012 standard.

Moreover, the publication of the evaluation reports, performed under international consensus documents and carried out by third-party bodies with respect to manufacturers, can also be useful in guiding the choice of laboratories in the introduction of new methods or instrumentation, as well as representing a paradigm for the execution of internal evaluation processes.

The ISO15189 standard does not specify which performance characteristics are to be assessed; the choice is left to the laboratory based on the method being verified. In general, the evaluation of accuracy and precision and the comparison of results with a reference method can be considered the parameters required to define the minimum analytical performances of a method [3,4].

In the field of haemostasis disorders, clinical laboratory plays an invaluable role in diagnostics and therapeutics. Thus, the availability of fast and inexpensive tests that meet the criteria of accuracy and precision required in the clinical laboratory is of great significance [5,6,7,8,9,10]. To this end, first-line screening tests, including activated partial thromboplastin time (APTT), prothrombin time (PT), fibrinogen (FIB), and the comprehensive screening test for platelet function, should meet the above requirements and should be characterised by a high negative predictive value [8,11,12,13,14].

Nowadays, automated coagulation analysers are highly available for clinical laboratories. However, coagulometers have become more complex; therefore, the evaluation process can be cumbersome and time-consuming. In addition, the specificity of reagents, often closely related to instruments, particular algorithms for data analysis, and, finally, different definitions of clot end-points, may influence clinical interpretation [15]. In this general context, differences in results are expected.

In addition, the mechanism of clot detection, whether photo-optical, mechanical, or hybrid, must be considered to direct the choice of the instrument. For example, it is commonly believed that the mechanical clot detection mode is not affected by interferents as, for example, in lipemic and haemolysed samples or hyperbilirubinemia [16]. However, some studies have demonstrated equivalence in terms of comparability, precision, and accuracy of optical and mechanical clotting analysers in routine analysis, even in the presence of sample turbidity [5,17,18,19].

This article summarises our technical evaluation of the Biolabo Solea 100 optical coagulometer in terms of accuracy and precision. A comparison with the Stago STA-R MAX mechanical coagulometer was also performed. Interference from triglycerides, haemoglobin, bilirubin, and heparin was also evaluated. 

## 2. Materials and Methods

### 2.1. Guidelines for the Evaluation Process

The evaluation process was performed in agreement with the Clinical and Laboratory Standards Institute guidelines [20,21,22,23,24].

### 2.2. Analyser Description

Biolabo Solea 100 (Biolabo SAS, Maizy, France) is a fully automated coagulation analyser that uses an optical system to detect coagulation. It is designed to meet the needs of medium and small laboratories. As stated by the manufacturer, the analyser has 8 readout channels able to analyse 110 samples per hour to determine the prothrombin time (PT) alone and 100 tests per hour for the panel prothrombin time, activated partial thromboplastin time (APTT), fibrinogen (FIB), and thrombin time (TT). In addition, the analyser can also determine D-Dimer, antithrombin III, protein C, and protein S. Chromogenic and immunological assays are performed at two wavelengths (405 nm and 620 nm). The analyser has a capacity of 32 samples, 16 reagent positions, 8 control and calibrator positions, and a cuvette rack loader that holds cuvettes for 464 tests.

The temperature in the cuvette, at a filling volume of 220 µL and 3 min waiting period, is kept constant at 37 °C ± 0.8 °C, while the temperature of the reagents is kept in a range between 16 °C and 22 °C, considering an operating temperature comprises between 17 °C and 28 °C.

Biolabo Solea 100 has an optomechanical measuring system and comprises disposable cuvettes containing a metal ball that allows the plasma sample and reagent to be mixed through a tilting motion. In addition, the rotation of the sphere encourages the aggregation of fibrin around the sphere. Aggregation is, however, detected through a change in light transmission using an optical system.

### 2.3. Reagents and Control Materials

The reagents used on SOLEA 100 were from Biolabo (Maizy, France), specifically BIO-CK APTT cephalin kaolin (APTT), BIO-FIBRI determination of fibrinogen (FIB), and BIO-TP Prothrombin Time (PT). Reagents used for STA-R were from STAGO Diagnostica (Asnières sur Seine, France), specifically STA-Cephascreen (APTT), STA-Liquid FIB (FIB), and STA-Neoplastine CI plus (PT). Commercially available lyophilised plasma samples were all from Biolabo: COATROL 1 and Control Plasma (CP) level 1 for normal values of all coagulation parameters; COATROL 2 for the low pathological value of FIB; Control Plasma level 2 for the medium pathological value of PT; Control plasma level 3 for the high pathological values of APTT and PT. Lipofundin MCT was from B-Braun Melsungen AG (Milan, Italy), bilirubin and low-molecular-weight (LWM) heparin from Sigma-Aldrich (Milan, Italy), and haemoglobin from liquichek haematology trilevel minipack Biorad (Milan, Italy).

### 2.4. Patient Samples

Blood samples were randomly selected from consecutive patient samples during the daily routine of the clinical laboratory for standard coagulation. The Clinical Laboratory is part of the tertiary care Regional Hospital “San Salvatore”. Blood samples were collected in a first pilot tube, then discarded, followed by a second evacuated tube (Vacutainer, Becton Dickinson Medical, Milan, Italy) containing 3.2% sodium citrate. Plasma was obtained by centrifugation at 4000× *g* rpm (1500× *g*) for 10 min at 20 °C and analysed within 4 h of collection. All samples were analysed for APTT (200), FIB (181), and FIB (206). 

This study was conducted on otherwise discarded anonymous specimens collected at the local hospital during daily laboratory activities. According to the Helsinki Declaration and FDA guidelines on informed consent for in vitro diagnostic device studies using leftover human specimens, patient informed consent was not applied because anonymous leftover material is usually destroyed. The Internal Review Board of the University of L’Aquila approved the study (IRB reference 26/2020)

### 2.5. Comparability Testing

Comparability testing experiments were executed according to the guidelines CLSI EP09-A3 “Measurement procedure comparison and bias estimation using patient samples” [9]. The results obtained from the Solea 100 were compared with those obtained from the STA-R MAX: a total of 200 samples for APTT, 181 for FIB, and 206 for PT, covering the broadest possible range, were analysed on both analysers simultaneously. The agreement of data generated by the two coagulation analysers was assessed with the Bland–Altman method of difference plot, Spearman’s correlation, and Passing–Bablock regression analysis. The application of Passing–Bablok linear regression was verified with the Cusum test for linearity [23,25,26]. A small *p*-value (*p* < 0.05) indicated no linear relationship between the two measurements; therefore, the Passing–Bablok method was not applicable. Outliers were detected using the Tukey’s test, while normal distribution was ascertained with the D’Agostino–Pearson test (*p* < 0.05). However, no outliers were identified. 

The bias at specific medical decision levels was calculated by standard bootstrap CI approximation for each coagulation parameter, imposing 1000 bootstrapping replications according to CLSI guideline EP09-A3 [23]. Results are shown in the Supplementary Material Tables S1–S3 for APTT, FIB, and PT, respectively.

The concordance correlation coefficient (ρ_c_) was calculated as described by Lin and used to evaluate the degree of concordance of pairs of observations that fall on the identity line [27]. The ρ_c_ is the precision (ρ), calculated as Pearson’s correlation coefficient, multiplied by the accuracy (C_b_), which measures how far the fitted line deviates from the line of identity. The strength of agreement can be extrapolated from the ρ_c_ value as follows: <0.90, poor; 0.90–0.95, moderate; 0.95–0.99, substantial; and >0.99, almost perfect.

### 2.6. Imprecision Studies

Imprecision in terms of repeatability was assessed in normal and pathological lyophilised plasmas consecutively 20 times in one run. All samples were analysed within 4 h of reconstitution. Intermediate imprecision was measured by analysing the same control plasmas for 10 days, twice daily, within 3 and 4 h from reconstitution. All experiments were performed as described by CLSI guidelines EP15-A3 [24]. In addition, fresh control samples of the same lot number were reconstituted daily.

Bias was determined for each control plasma by comparing the mean value obtained from the intermediate repeatability assay with the value claimed by the manufacturer. 

The total error (TE) was estimated by the imprecision (I) obtained from the intermediate repeatability experiment and the bias (B) calculated as previously described by Ricos et al. [28]:

TE(%)=(1.65×I)+B

As Gardiner suggested for routine coagulation assays, the total allowable error (TAE) was calculated using the above equation setting a flat limit at 3% for imprecision and bias [15]. Thus, the internal criterion TAE was 7.95%, whereas the manufacturer’s TAE is based on the GEHT recommendations (Groupe Français d’Étude sur l’Hémostase et la Thrombose, Normes d’acceptabilité en hémostase, 2014). 

### 2.7. Interference Testing

Interference on the determination of APTT, FIB, and PT values was evaluated according to CLSI EP07-A3 [22] in a dose–response experiment in the presence of increasing concentrations of triglycerides, haemoglobin, bilirubin, and fractionated low-molecular-weight heparin as previously described with some modification [29,30,31,32]. Lipofundin MCT (B. Braun-Melsungen AG, Melsungen, Germany) is a 20% emulsion of nonpolar lipids (10% soybean oil and 10% medium chain triglycerides). Control plasmas were spiked with increasing concentrations of Lipofundin MCT to achieve nominal concentrations of triglycerides ranging from 0.68 mmol/L to 7.57 mmol/L. 

Haemolysed Liquichek Hematology trilevel (BioRad, Milan, Italy) was used to reproduce haemolysed plasma. Briefly, the blood was centrifuged at 2500× *g* rpm for 10 min at 18 °C. The supernatant was discarded, and the pellet was resuspended in a 0.9% NaCl solution to a 1:10 ratio and then centrifuged. The process was repeated three times. The supernatant was discarded, and distilled water was added to induce cell haemolysis. After incubation at 4 °C for 15 min, the solution was centrifuged, and the supernatant was recovered for haemoglobin determination. Normal and pathological plasmas were spiked with haemoglobin solution at concentrations ranging from 0.32 g/L to 4.2 g/L.

Normal and pathological control plasmas were spiked with increasing concentrations of commercial bilirubin to reproduce icteric plasma samples (Sigma-Aldrich, Milan, Italy), obtaining nominal bilirubin concentrations ranging from 25 µmol/L to 370 µmol/L. 

Low-molecular-weight heparin (Sigma Aldrich, Milan, Italy), ranging from 0.3 IU/mL to 2.0 IU/mL, was added to the control plasmas.

The criteria of acceptability were defined using the following equation, according to Fraser [33]:

$$\mathrm{CA}=1.96\times \sqrt{{\left({\mathrm{CV}}_{a}\right)}^{2}+{\left({\mathrm{CV}}_{w}\right)}^{2}}$$

where the analytical imprecision (CV_a_) was obtained from the noninterfered samples, calculated as the standard deviation over the mean of the measurements, and the within-subject biological variation (CV_w_), as reported by Ricos [28]. 

All experiments were performed in triplicate at each concentration of tested interferent.

### 2.8. Software

Statistical analysis was performed using MedCalc version 18.2.1 (MedCalc Software, Ostend, Belgium) and OriginPro version 8.5.1 (OriginLab Corporation, Northampton, MA, USA).

### 2.9. Operating Temperature

All tests were performed by constantly monitoring the ambient temperature between 23 °C and 28 °C.

## 3. Results

### 3.1. Comparability Testing

**Figure 1:**
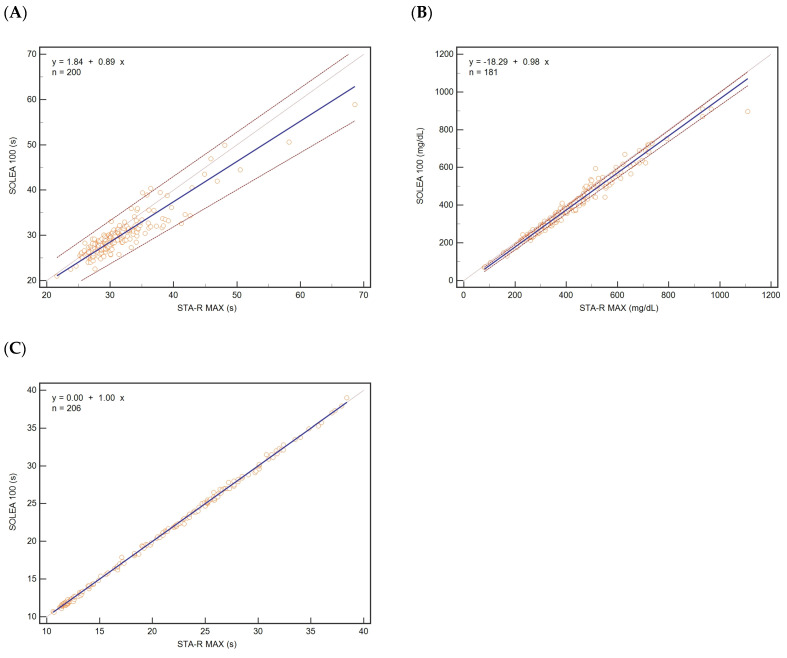
PassingBablok regression analysis for (**A**) activated partial thromboplastin time (APTT), 200 samples; (**B**) fibrinogen (FIB), 181 samples; and (**C**) prothrombin time (PT), 206 samples to evaluate the correlation between STA-R MAX (x-axis) and Solea 100 (y-axis). The solid line represents the regression line, the dotted line the theoretical identity line, and the dashed lines the 95% confidence interval.

**Figure 2:**
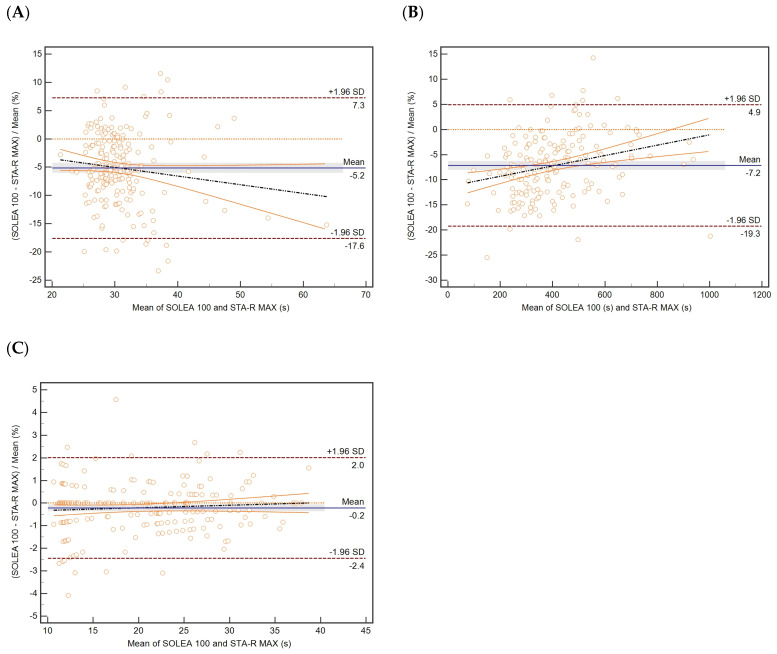
BlandAltman difference plots for (**A**) activated partial thromboplastin time (APTT), 200 samples; (**B**) fibrinogen (FIB), 181 samples; and (**C**) prothrombin time (PT), 206 samples to evaluate the correlation between STA-R MAX and Solea 100. The solid line represents the mean percentage difference, the dotted line the theoretical identity line, the dashed lines the 95% confidence interval, the dot-dashed line the regression line, the solid orange line the 95% CI of the regression line, and the grey band the 95% confidence interval on the mean percentage difference value.

The comparison study between Biolabo Solea 100 and Stago STA-R was carried out by analysing plasma samples for APTT (200 samples), FIB (181 samples), and PT (206 samples), covering the most extensive possible range on both instruments. The results of the study are shown in Table 1. The data obtained were analysed by linear Passing–Bablok regression (Table 1 and Figure 1), by Bland–Altman plot (Figure 2), and by determination of the correlation coefficient of concordance (Table 2).

For APTT, a slight deviation from the identity line (slope of 0.89) can be observed. A systematic deviation of about 2 s (Table 1, Figure 1A) and a negative bias of about −5% (Figure 2A) are observed. The estimated correlation coefficient for APTT has a magnitude barely less than 0.9. 

The comparison analysis of the results for the FIB is satisfactory. The slope of the regression line is close to 1 with a narrow 95% confidence interval. A constant negative systematic deviation of about −18 mg/dL is observed (Table 1, Figure 1B), equivalent to an approximately −7% bias (Table 1, Figure 2B). The Spearman’s rank correlation coefficient is close to 1 (0.989) (Table 1). 

The best correlation result was obtained for the PT value. For instance, no proportional or systematic deviations from the theoretical identity line can be observed. The slope and intercept values of the regression line are equal to 1 and 0, respectively (Table 1, Figure 1C). For this parameter, the correlation coefficient is close to 1 (0.997) (Table 1), and the average bias value determined by the Bland–Altman method is close to 0 (Table 1, Figure 2C). 

The applicability of the Passing–Bablock analysis method was ascertained with the CUSUM test for linearity, which is commonly used to evaluate the linear relationship among the results of the reference methods and those tested. For ATPP, FIB, and PT, the *p*-value was greater than 0.05 (APTT, *p* = 0.8; FIB, *p* = 0.5; and PT, *p* = 0.9).

The calculated concordance correlation coefficients (ρ_c_) are shown in Table 2 and are consistent with the Passing–Bablock analysis. The strength of agreement is poor in the case of APTT, with a concordance coefficient value less than 0.90 (ρ_c_ = 0.8682), while it is substantial for FIB (ρ_c_ = 0.9721). A high level of concordance between the two analysers is confirmed for PT, with a concordance coefficient value nearly equal to 1 (ρ_c_ = 0.9996), which corresponds to an almost perfect strength of agreement. 

The bootstrap technique was used to assess the bias at specific medical decision levels (Tables S1–S3 Supplementary Materials). As shown in Table S1, the APTT shows a negative relative percentage difference of approximately −6% at the normal medical decision level, which becomes approximately −9% at the pathological level of 90 s. The same trend can be observed for FIB, although with larger relative difference values. The difference at the normal medical decision level (500 mg/dL) is around −5%, which progressively increases in the proximity of the pathological limits, reaching a value of −63% at the medical level of 30 mg/dL (Table S2). Concerning PT (Table S3), the relative difference is zero at all medical decision levels.

### 3.2. Imprecision Studies

The results of repeatability and intermediate imprecision are reported in Table 3. For each of the measured parameters, the value of imprecision, expressed as CV% and bias%, was reported, and they were compared with the flat limit of 3% chosen as an acceptability criterion. 

Overall, the coagulation parameters, normal and pathological levels, showed a total error below the acceptability criterion of 7.95%, except for the pathological control of FIB, whose total error was calculated at 10.77%.

### 3.3. Interference Testing

No interferences were observed for nominal concentrations of triglycerides up to 7.9 mmol/L (approximately 8.6 mmol/L for total plasma triglycerides) and 4.2 g/L of haemoglobin for all tests (Figures S1 and S2 in the Supplementary Materials). Indeed, the bias for all coagulation values lies perfectly within the imposed 5% acceptability criterium. 

In the case of the coagulation parameter of APTT, bias values above the imposed acceptability limit of 5.4% were observed at nominal bilirubin values greater than 100 µmol/L for normal control and greater than 250 µmol/L for the pathological control (Figure S3A,B). On the other hand, no interferences were observed for FIB in the normal and pathological control plasmas when icteric samples were simulated (Figure S3C,D). 

The bias measured for the PT parameter in the presence of increasing bilirubin concentrations is outside the acceptable limits for bilirubin concentrations greater than 250 µmol/L for the normal control plasma and 375 µmol/L for the pathological control plasma (Figure S3E,F).

There was no interference for fibrinogen in the heparinised samples, where the observed biases were less than 5% in the normal and pathological plasmas (Figure S4A,B). 

As stated by the manufacturer, no interference in PT determination is observed for heparin concentrations below 0.11 IU/mL. This is true for normal plasma, where no interference is observed at 0.3 IU/mL. In contrast, at 0.4 IU/mL, the bias is 8.8%, a few decimal places above the acceptability limit of 8.0% (Figure S4C). For pathological plasma, interference is observed at 0.3 IU/mL with a bias of approximately 16%, twice the limit of acceptability (Figure S4D). The APTT value could not be determined in the presence of heparin.

## 4. Discussion

In this study, we evaluated the first-line screening tests, which include activated partial thromboplastin time (APTT), fibrinogen (FIB), and prothrombin time (PT), routinely performed in the assessment of the coagulation status.

The analytical performance of the analyser was measured in terms of comparison of the results obtained by measuring the analytical parameters in human samples using the STAGO STA-R MAX mechanical coagulometer as the reference method. The results of the comparison experiment are shown in Table 1. Among these, the most impressive results are those relating to the determination of the PT. The values obtained by the two analysers are perfectly comparable and lie globally on the theoretical identity line. The Spearman’s correlation coefficient and the concordance coefficient also confirmed the high level of agreement between the two analysers.

The same level of agreement was observed in the determination of FIB, as also confirmed by the Spearman’s correlation and concordance coefficients, although a systematic underestimation was observed. 

In contrast, the comparative results for APTT are less satisfactory when compared with FIB and PT. Although the data are on the verge of lying on the identity line, and no evidence of gross bias is observed, a low level of concordance is estimated. However, from a comparison of the results of this study with other studies, it is possible to realise that the results are comparable with higher profile instrumentations.

In a study by Lippi et al., the Roche Cobas t711 (Roche Diagnostics GmbH) is compared with the Instrumentation Laboratory ACL TOP 700 (Instrumental Laboratory) and Stago STA-R MAX (Stago Diagnostics SAS). In this study, the three instruments are also compared with each other [5]. The comparison shows Spearman’s correlation coefficients comparable to those obtained in this study for the Biolabo Solea 100: for PT, it is about 0.97; for FIB, it is between 0.95 and 0.97; and for APTT, it is between 0.74 and 0.88 [5]. It should also be considered that the regression lines estimated for PT show slope values that are often far from the theoretical value: 0.78 for Cobas t711/ACL TOP comparison, 0.61 Cobas t711/STA-R MAX, and 1.25 for ACL TOP/STA-R MAX.

The repeatability, the intermediate imprecision, and the bias were also determined. Compared with the data stated by the manufacturer (Table 3), this study shows biases, calculated on repeatability and intermediate imprecision, comparable to, or even lower than, those declared. An exception is the FIB, where the calculated repeatability for normal and pathological control plasmas is higher than those calculated by the manufacturer in the repeatability experiment, as well as for normal plasma in intermediate imprecision. Nevertheless, the results from this study in terms of imprecision and bias are generally consistent with those obtained in other studies [5,17,34].

The results obtained from this study in terms of interference are approximately in line with the manufacturer’s statement for all tests. However, it should be considered that the method leaflets do not contain the experimental conditions of the interference tests and the defined criteria of acceptability. The manufacturer claims no interference for APTT determination up to a turbidity value of 0.543 O.D., positive interference above a bilirubin concentration of 143 µmol/L, and no interference in haemolytic samples up to a haemoglobin concentration of 261 µmol/L. According to the manufacturer, FIB determination is not affected by turbidity with optical density values below 0.543 O.D., haemoglobin up to 261 µmol/L (4.2 g/L), bilirubin up to 496 µmol/L, and low-molecular-weight heparin below 2.0 IU/L. 

Concerning the evaluation of the effects of interferents on PT determination, the manufacturer’s package insert states no interference with sample turbidity up to 0.390 O.D., no interference from haemoglobin up to concentrations of 258 µmol/L (about 4.2 g/L), and positive interference at bilirubin concentrations starting from 171 µmol/L and low-molecular-weight heparin at 0.11 IU/mL. Therefore, it is impossible to directly correlate the turbidity expressed in optical density with the concentration of triglycerides. Nevertheless, this study shows that even at the highest triglyceride concentration tested, the measurement of the coagulation parameters is not influenced.

Although with some differences, the results are consistent with those described by Nougier et al. for the ACLTOP-750 and STA-Compact MAX coagulometers in lipemic and homolysed samples [34]. There were no differences between the two studies in lipemic samples, limited to the highest triglyceride concentration tested. A significant bias for both instruments was described for haemoglobin values above 5 g/L for PT and approximately 2 g/L for APTT and FIB. However, in jaundiced samples, SOLEA 100 performs worse than ACLTOP-750 and STA-compact MAX, as a significant bias is observed at bilirubin values above 513 µmol/L. However, the interference exerted by bilirubin is comparable with the manufacturer’s statement. 

## 5. Conclusions

The verification of the analytical performance of a method or instrumentation is one of the fundamental aspects of quality control of the entire analytical process, ensuring, along with cost containment and the ability to rapidly provide a reliable answer, the efficacy of the clinical laboratory. It is precisely in this context of the efficiency of the action of the clinical laboratory that this study is placed.

In this study, we summarised our technical evaluation of the Biolabo Solea 100 optomechanical coagulometer in terms of accuracy and precision, comparability of results with the Stago STA-R MAX mechanical coagulometer, and interferences of triglycerides, haemoglobin, bilirubin, and heparin.

Many other aspects should have been investigated and considered, such as the influence of the used surface on platelet activation, the scaling with fibrinogen concentration, platelet count, and the stability on board the reagents.

However, the preliminary performance evaluation of the Biolabo SOLEA 100 automatic haemostasis analyser and Biolabo reagents for APTT, FIB, and PT are comparable to those obtained from the Stago STA-R MAX instrument in terms of comparison. Furthermore, the reference ranges, imprecision, and the effect of common interferents agree with the manufacturer’s statements. 

In summary, the results are satisfactory, and the instrument can be considered for routine use because it meets the criteria of acceptability.

## Figures and Tables

**Table 1:** Comparison among methods for activated partial thromboplastin time, fibrinogen, and prothrombin time.

		Median (Ranges)					
Parameter *^a^*	Sample Size	STA-R	SOLEA 100	Slope (95% CI)	Intercept (95% CI)	BIAS (%) (95% CI)	Correlation Coefficient *^b^* (95% CI)
APTT (s)	200	30.4 (21.6 to 68.6)	29.1 (21.0 to 58.9)	0.89 (0.82 to 0.98)	1.84 (−0.84 to 3.99)	−5.16 (−6.05 to −4.28)	0.874 (0.837 to 0.903)
FIB (mg/dL)	181	388.0 (80.0 to 1109.0)	368.0 (69.0 to 910.0)	0.98 (0.96 to 1.01)	−18.29 (−29.23 to −9.76)	−7.16 (−8.07 to −6.26)	0.989 (0.985 to 0.992)
PT (s)	206	18.8 (10.6 to 39.0)	18.9 (10.6 to 38.4)	1.00 (1.00 to 1.00)	0.00 (0.00 to 0.00)	−0.21 (−0.37 to −0.06)	0.997 (0.996 to 0.997)

Results from Stago STA-R MAX and Biolabo SOLEA 100 are compared using the Passing–Bablok method. *^a^* Activated partial thromboplastin time (APTT), fibrinogen (FIB), and prothrombin time (PT). *^b^* The Spearman’s rank correlation coefficient.

**Table 2:** Comparison among methods for activated partial thromboplastin time, fibrinogen, and prothrombin time.

Parameter *^a^*	Sample Size	Pearson’s Correlation Coefficient (*ρ*)	Bias Correction Factor (*C*_b_)	Concordance Coefficient (*ρ*_c_) (95% CI)	Strength of Agreement *^b^*
APTT (s)	200	0.9180	0.9458	0.8682 (0.8334 to 0.8962)	poor
FIB (mg/dL)	181	0.9857	0.9863	0.9721 (0.9636 to 0.9787)	substantial
PT (s)	206	0.9996	1.000	0.9996 (0.9994 to 0.9997)	almost perfect

Results from Stago STA-R MAX and Biolabo SOLEA 100 are compared by the concordance correlation coefficient method. *^a^* Activated partial thromboplastin time (APTT), fibrinogen (FIB), and prothrombin time (PT). *^b^* The strength of agreement can be extrapolated from the *ρ*_c_ value as follows: <0.90, poor; 0.90–0.95, moderate; 0.95–0.99, substantial; and >0.99, almost perfect.

**Table 3:** Results of imprecision and bias studies on Biolabo Solea 100.

			Repeatability		Intermediate Imprecision			Internal Criteria *^b^*		SOLEA 100	
	Sample *^a^*	Target Value	CV%	BIAS%	CV%	BIAS%	Calculated TE%	TAE%	Within-Run *^c^* CV%	Between-Run *^c^* CV%	TAE% *^d^*
**APTT (s)**	COATROL 1 (normal)	35.00	0.90	2.57	2.89	0.60	5.37	7.95	0.9	2.9	25.04
	CP level 3 (high)	60.00	0.40	3.00	**4.40**	0.52	7.78	7.95	0.7	4.4	22.36
**FIB (mg/dL)**	CP level 1 (normal)	343.00	**4.50**	−0.12	**3.41**	−1.46	7.08	7.95	3.3	2.7	23.94
	COATROL 2 (low)	143.00	**3.50**	**−6.99**	2.74	**4.41**	**10.77**	7.95	2.1	5.1	41.93
**PT (s)**	CP level 1 (normal)	12.60	0.82	−0.48	1.25	0.24	2.30	7.95	1.5	1.9	12.99
	CP level 2 (medium)	21.50	0.97	−0.05	**3.08**	0.19	5.27	7.95	1.6	4.2	20.27
	CP level 3 (high)	30.00	1.22	0.17	2.04	1.50	4.87	7.95	1.9	3.1	39.90

*^a^* COATROL 1 and Control Plasma (C.P.) level 1 for within-range concentrations of all parameters, COATROL 2 for low pathological concentration of FIB, Control Plasma level 2, and Control plasma level 3 for low pathological concentrations of APTT and P.T. *^b^* TAE was calculated using a rule of thumb 3% for imprecision and bias. In bold data with values higher than 3% or higher than the TAE. *^c^* Within-run and between-run imprecisions stated by the manufacturer. *^d^* Criteria based on the recommendations of the GEHT (Groupe Français d’Étude sur l’Hémostase et la Thrombose, Normes d’acceptabilité en hémostase, 2014).

## Data Availability

Not applicable.

## Supplementary Materials

The following supporting information can be downloaded at: https://www.mdpi.com/article/10.3390/diagnostics13010085/s1, Table S1. APTT: bias at specific decision levels, obtained by standard bootstrap CI approximation, calculated by imposing 1000 bootstrap replications. Table S2. FIB: bias at specific decision levels, obtained by standard bootstrap CI approximation calculated by imposing 1000 bootstrap replications. Table S3. PT: bias at specific decision levels, obtained by standard bootstrap CI approximation calculated by imposing 1000 bootstrap replications. Figure S1. Plot of the bias as a function of the concentration of triglycerides. Figure S2. Plot of the bias as a function of the concentration of haemoglobin. Figure S3. Plot of the bias as a function of the concentration of bilirubin. Figure S4. Plot of the bias as a function of the concentration of low-molecular-weight (LMW) heparin.

## References

[1] Plebani M. (2018). Quality and Future of Clinical Laboratories: The Vico’s Whole Cyclical Theory of the Recurring Cycles. Clin. Chem. Lab. Med..

[2] (2012). Medical Laboratories—Requirements for Quality and Competence.

[3] Antonelli G., Sciacovelli L., Aita A., Bozzato D., Plebani M. (2019). The Pathway for Introducing Novel Examination Procedures in Routine Practice in Accordance with ISO 15189:2012: 17-Hydroxy Progesterone, Dehydroepiandrosterone Sulphate and Vitamin D as Examples. Ann. Clin. Biochem..

[4] Antonelli G., Padoan A., Aita A., Sciacovelli L., Plebani M. (2017). Verification of Examination Procedures in Clinical Laboratory for Imprecision, Trueness and Diagnostic Accuracy According to ISO 15189:2012: A Pragmatic Approach. Clin. Chem. Lab. Med..

[5] Lippi G., Salvagno G., Gelati M., Poli G., Giavarina D., Favaloro E. (2019). Analytical Assessment of the New Roche Cobas t 711 Fully Automated Coagulation Analyzer. Semin. Thromb. Hemost..

[6] Zantek N.D., Hayward C.P., Simcox T.G., Smock K.J., Hsu P., Van Cott E.M. (2016). An Assessment of the State of Current Practice in Coagulation Laboratories. Am. J. Clin. Pathol..

[7] Bennett S.T., Lehman C.M., Rodgers G.M. (2015). Laboratory Hemostasis.

[8] Lippi G., Favaloro E.J. (2018). Laboratory Hemostasis: From Biology to the Bench. Clin. Chem. Lab. Med..

[9] Theodorsson E., Magnusson B. (2017). Full Method Validation in Clinical Chemistry. Accredit. Qual. Assur..

[10] Marlar R.A., Gausman J.N., Engel J.W. (2014). Validation of Hemostasis and Coagulation Assays: Recommendations and Guidelines. Semin. Thromb. Hemost..

[11] Bonar R.A., Lippi G., Favaloro E.J. (2017). Overview of Hemostasis and Thrombosis and Contribution of Laboratory Testing to Diagnosis and Management of Hemostasis and Thrombosis Disorders. Methods in Molecular Biology.

[12] Favaloro E.J., Lippi G., Adcock D.M. (2008). Preanalytical and Postanalytical Variables: The Leading Causes of Diagnostic Error in Hemostasis?. Semin. Thromb. Hemost..

[13] Gardiner C., Coleman R., de Maat M.P.M., Dorgalaleh A., Echenagucia M., Gosselin R.C., Ieko M., Kitchen S. (2021). International Council for Standardization in Haematology (ICSH) Laboratory Guidance for the Evaluation of Haemostasis Analyser-Reagent Test Systems. Part 1: Instrument-Specific Issues and Commonly Used Coagulation Screening Tests. Int. J. Lab. Hematol..

[14] Adcock Funk D.M., Lippi G., Favaloro E.J. (2012). Quality Standards for Sample Processing, Transportation, and Storage in Hemostasis Testing. Semin. Thromb. Hemost..

[15] Gardiner C., Kitchen S., Dauer R., Kottke-Marchant K., Adcock D. (2006). Recommendations for Evaluation of Coagulation Analyzers. Lab. Hematol..

[16] Flanders M.M., Crist R., Safapour S., Rodgers G.M. (2002). Evaluation and Performance Characteristics of the STA-R Coagulation Analyzer. Clin. Chem..

[17] Geens T., Vertessen F., Malfait R., Deiteren K., Maes M.B. (2015). Validation of the Sysmex CS5100 Coagulation Analyzer and Comparison to the Stago STA-R Analyzer for Routine Coagulation Parameters. Int. J. Lab. Hematol..

[18] Bai B., Christie D.J., Gorman R.T., Wu J.R. (2008). Comparison of Optical and Mechanical Clot Detection for Routine Coagulation Testing in a Large Volume Clinical Laboratory. Blood Coagul. Fibrinolysis.

[19] Fischer F., Appert-Flory A., Jambou D., Toulon P. (2006). Evaluation of the Automated Coagulation Analyzer Sysmex^®^ CA-7000. Thromb. Res..

[20] Clinical and Laboratory Standards Institute (2010). Validation, Verification, and Quality Assurance of Automated Hematology Analyzers.

[21] Clinical and Laboratory Standards Institute, Clinical and Laboratory Standards Institute (2008). Protocol for the Evaluation, Validation, and Implementation of Coagulometers.

[22] Clinical and Laboratory Standards Institute (2018). Interference Testing in Clinical Chemistry; Approved Guideline.

[23] Clinical and Laboratory Standards Institute (2013). Measurement Procedure Comparison and Bias Estimation Using Patient Samples; Approved Guideline.

[24] Clinical and Laboratory Standards Institute (2014). User Verification of Precision and Estimation of Bias; Approved Guideline.

[25] Martin Bland J., Altman D. (1986). Statistical Methods for Assessing Agreement Between Two Methods of Clinical Measurement. Lancet.

[26] Passing H., Bablok W. (1983). A New Biometrical Procedure for Testing the Equality of Measurements from Two Different Analytical Methods. Clin. Chem. Lab. Med..

[27] Lin L.I.-K. (1989). A Concordance Correlation Coefficient to Evaluate Reproducibility. Biometrics.

[28] Ricós C., Alvarez V., Cava F., García-Lario J.V., Hernández A., Jiménez C.V., Minchinela J., Perich C., Simón M. (1999). Current Databases on Biological Variation: Pros, Cons and Progress. Scand. J. Clin. Lab. Investig..

[29] Nougier C., Jousselme E., Sobas F., Pousseur V., Négrier C. (2020). Effects of Hemolysis, Bilirubin, and Lipemia Interference on Coagulation Tests Detected by Two Analytical Systems. Int. J. Lab. Hematol..

[30] Nikolac N. (2014). Lipemia: Causes, Interference Mechanisms, Detection and Management. Biochem. Med..

[31] Krouwer J.S. (2012). Interference Testing: Why Following Standards Is Not Always the Right Thing to Do. J. Diabetes Sci. Technol..

[32] Nagant C., Rozen L., Demulder A. (2016). HIL Interferences on Three Hemostasis Analyzers and Contribution of a Preanalytical Module for Routine Coagulation Assays. Clin. Lab..

[33] Fraser C.G. (2004). Test Result Variation and the Quality of Evidence-Based Clinical Guidelines. Proc. Clin. Chim. Acta.

[34] Quehenberger P., Kapiotis S., Handler S., Ruzicka K., Speiser W. (1999). Evaluation of the Automated Coagulation Analyzer SYSMEX CA 6000. Thromb. Res..

