# Supplementary material for: Evaluation of the Analytical Performances of the Biolabo SOLEA 100 Optical Coagulometer and Comparison with the Stago STA-R MAX Analyser in the Determination of PT, APTT, and Fibrinogen
Source: Diagnostics (Basel). 2022 Dec 28;13(1):85. doi: 10.3390/diagnostics13010085 (PMC9818767; doi:10.3390/diagnostics13010085)

Supplementary material

Evaluation of the analytical performances of the Biolabo SOLEA 100 optical coagulometer and comparison with the Stago STA-R MAX analyser in the determination of PT, APTT and fibrinogen

Table S1. APTT: bias at specific decision levels, obtained by standard bootstrap CI approximation, calculated by imposing 1000 bootstrap replications.

Medical decision level[a]	Modelled Y (95% CI)	Difference (95% CI)	Relative difference (95% CI)
35	32.9837<br>32.4080 to 33.5288	-2.0163<br>-2.5920 to -1.4712	-5.76%<br>-7.41 to -4.20%
45	41.8833<br>40.5493 to 43.5000	-3.1167<br>-4.4507 to -1.5000	-6.93%<br>-9.89 to -3.33%
90	81.9311<br>76.8227 to 88.5000	-8.0689<br>-13.1773 to -1.5000	-8.97%<br>-14.64 to -1.67%

[a]Medical decision levels from Statland BE. Clinical Decision Levels for Laboratory Tests, Second Edition [Oradell NJ; Medical Economics Books, 1987.]

Table S2. FIB: bias at specific decision levels, obtained by standard bootstrap CI approximation calculated by imposing 1000 bootstrap replications.

Medical decision level[a]	Modelled Y (95% CI)	Difference (95% CI)	Relative difference (95% CI)
30	11.1786<br>2.0000 to 19.1868	-18.8214<br>-28.0000 to -10.8132	-62.74%<br>-93.33 to -36.04%
100	79.9286<br>72.0879 to 86.4466	-20.0714<br>-27.9121 to -13.5534	-20.07%<br>-27.91 to -13.55%
500	472.7857<br>466.7499 to 478.8600	-27.2143<br>-33.2501 to -21.1400	-5.44%<br>-6.65 to -4.23%

[a]Medical decision levels from Statland BE. Clinical Decision Levels for Laboratory Tests, Second Edition [Oradell NJ; Medical Economics Books, 1987.]

Table S3. PT: bias at specific decision levels, obtained by standard bootstrap CI approximation calculated by imposing 1000 bootstrap replications.

Medical decision level[a]	Modelled Y (95% CI)	Difference (95% CI)	Relative difference (95% CI)
14	14.0000<br>13.9250 to 14.0000	0.0000<br>-0.07500 to 0.0000	0.00%<br>-0.54 to 0.00%
16	16.0000<br>15.9250 to 16.0000	0.0000<br>-0.07500 to 0.0000	0.00%<br>-0.47 to 0.00%
30	30.0000<br>29.9049 to 30.0000	0.0000<br>-0.09515 to 0.0000	0.00%<br>-0.32 to 0.00%

[a]Medical decision levels from Statland BE. Clinical Decision Levels for Laboratory Tests, Second Edition [Oradell NJ; Medical Economics Books, 1987.]

Figure S1. Plot of the bias as a function of the concentration of triglycerides. (A) and (B) for APTT for normal plasma (COATROL1) and pathological plasma (Control Plasma level 3), respectively. (C) and (D) for FIB for normal plasma (Control Plasma level 1) and pathological plasma (COATROL 2), respectively. (E) and (F) for PT for normal plasma (Control Plasma level 1) and pathological plasma (COATROL 2), respectively. Solid black horizontal lines are the limits of acceptability. Data are reported as the mean of three independent determinations ± the standard error.

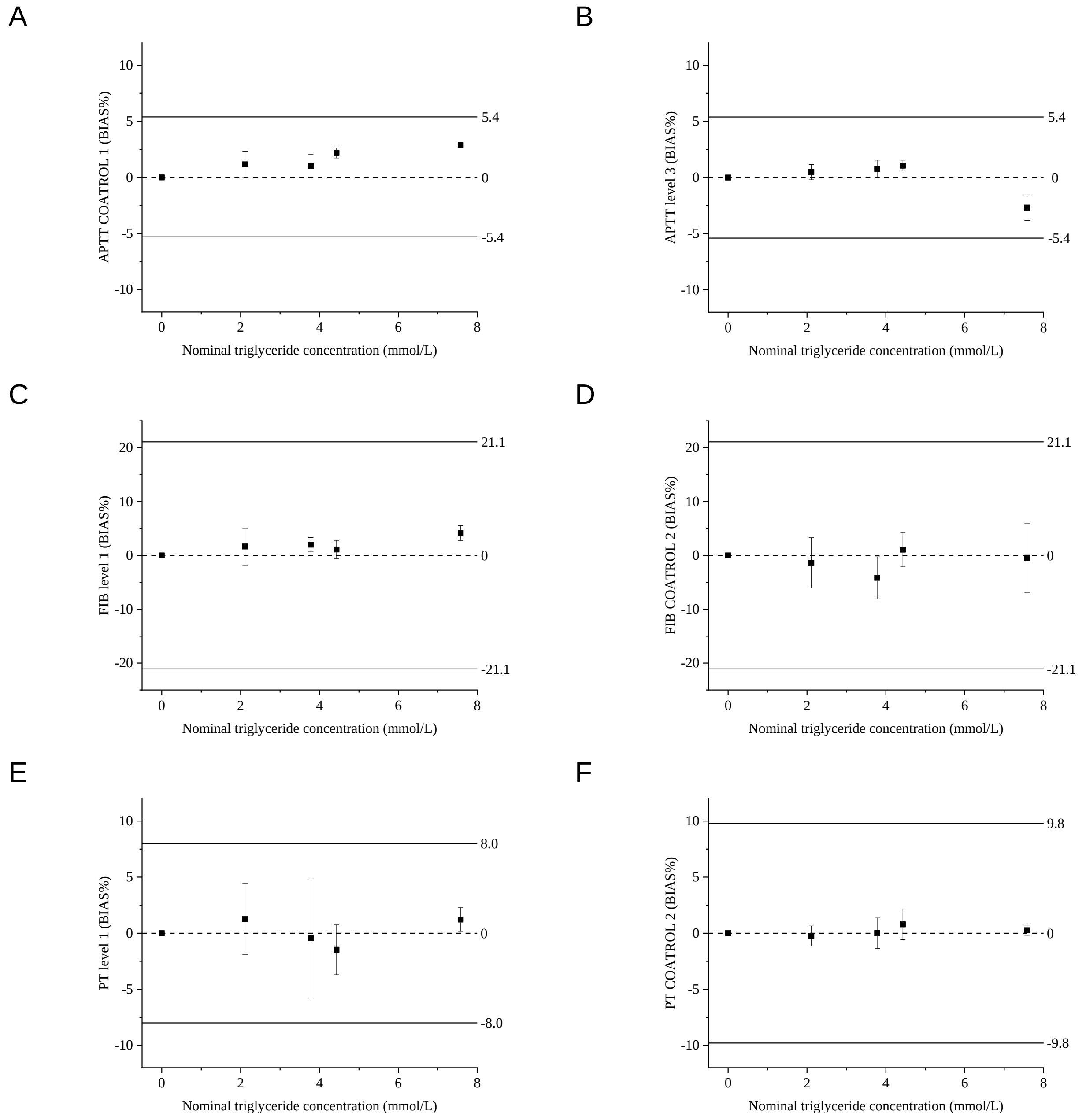

Figure S2. Plot of the bias as a function of the concentration of haemoglobin. (A) and (B) for APTT for normal plasma (COATROL1) and pathological plasma (Control Plasma level 3), respectively. (C) and (D) for FIB for normal plasma (Control Plasma level 1) and pathological plasma (COATROL 2), respectively. (E) and (F) for PT for normal plasma (Control Plasma level 1) and pathological plasma (COATROL 2), respectively. Solid black horizontal lines are the limits of acceptability. Data are reported as the mean of three independent determinations ± the standard error.

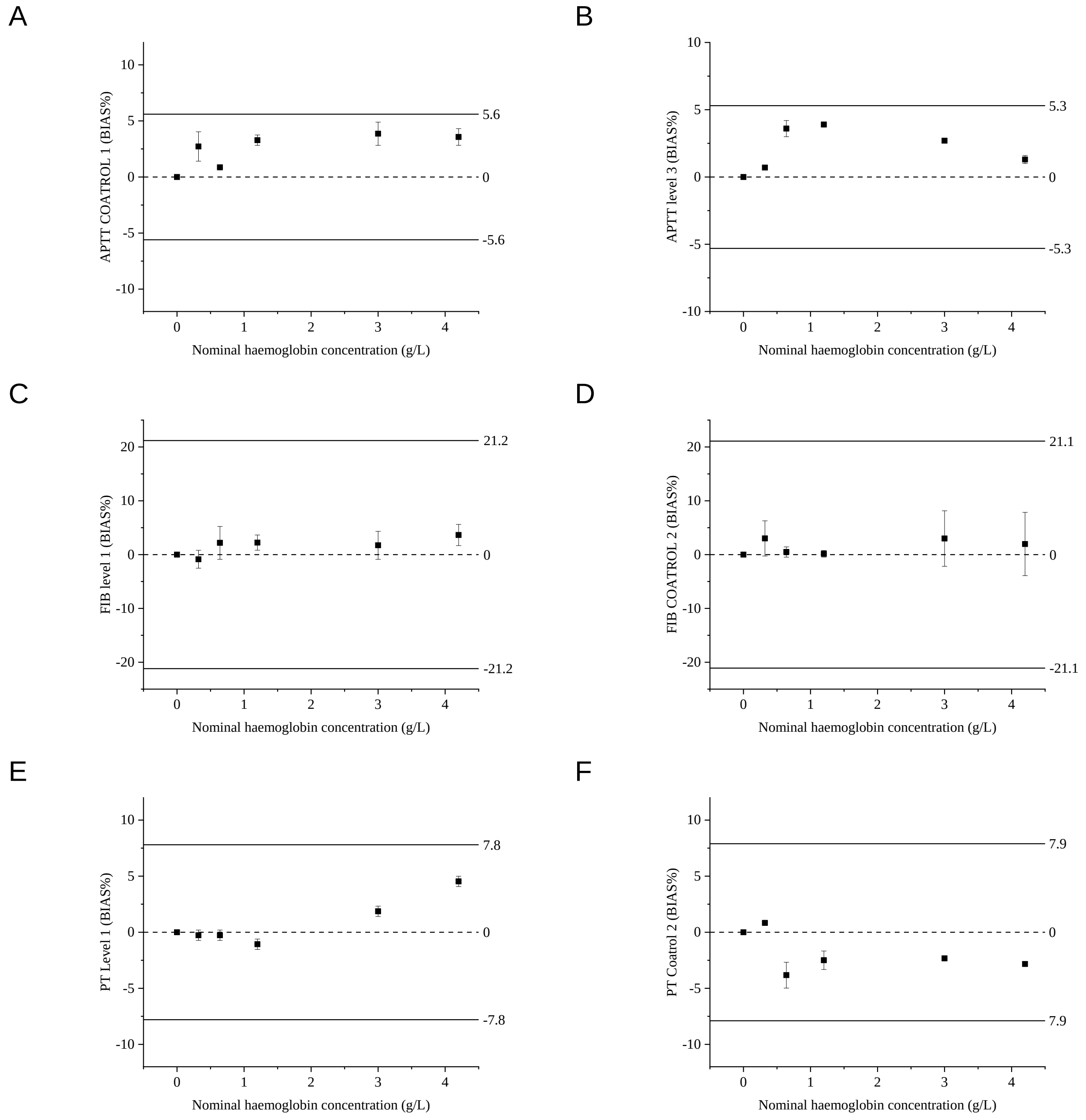

Figure S3. Plot of the bias as a function of the concentration of bilirubin. (A) and (B) for APTT for normal plasma (COATROL1) and pathological plasma (Control Plasma level 3), respectively. (C) and (D) for FIB for normal plasma (Control Plasma level 1) and pathological plasma (COATROL 2), respectively. (E) and (F) for PT for normal plasma (Control Plasma level 1) and pathological plasma (COATROL 2), respectively. Solid black horizontal lines are the limits of acceptability. Data are reported as the mean of three independent determinations ± the standard error.

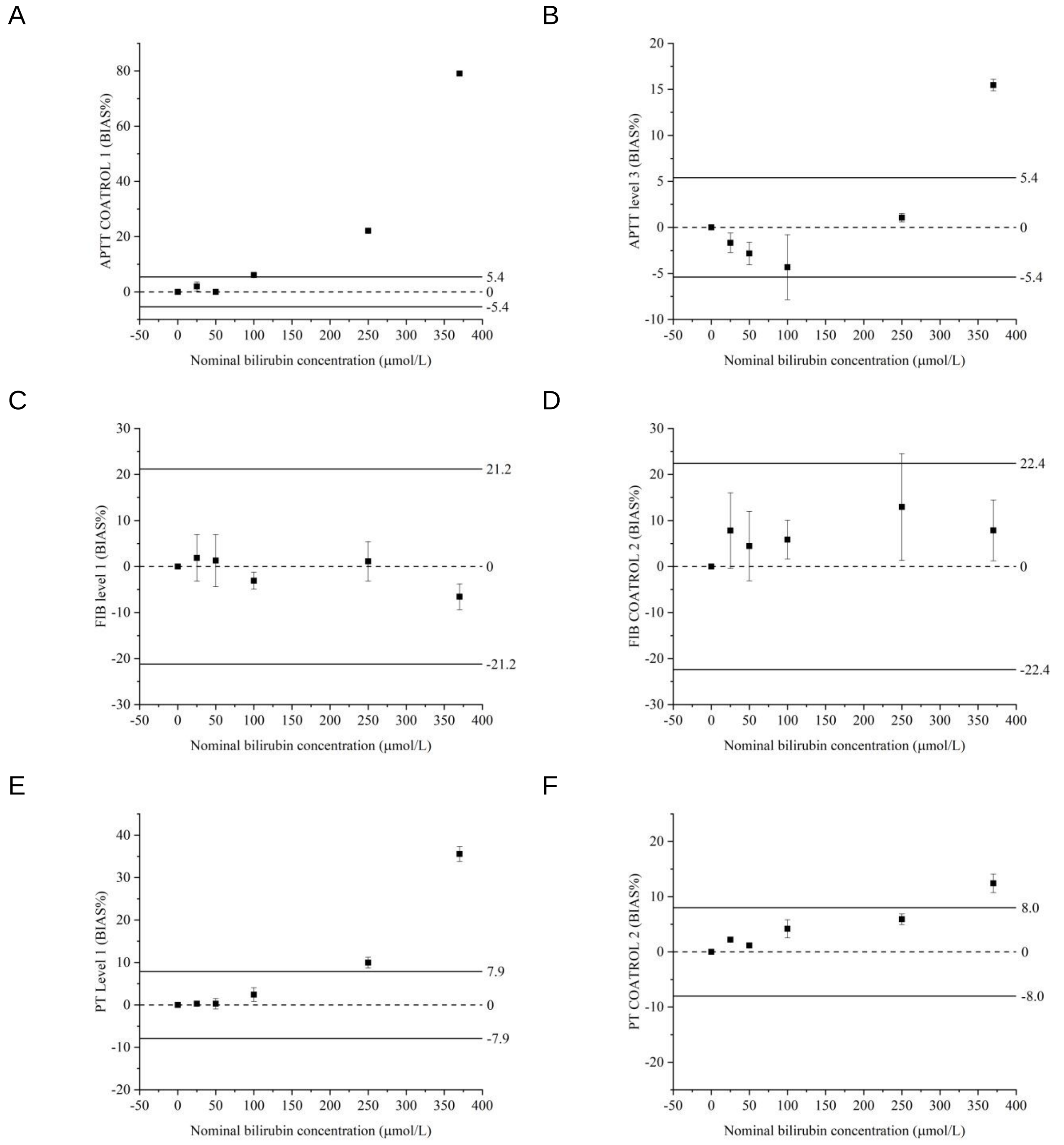

Figure S4. Plot of the bias as a function of the concentration of low molecular weight (LMW) heparin. (A) and (B) for FIB for normal plasma (Control Plasma level 1) and pathological plasma (COATROL 2), respectively. (C) and (D) for PT for normal plasma (Control Plasma level 1) and pathological plasma (COATROL 2), respectively. Solid black horizontal lines are the limits of acceptability. Data are reported as the mean of three independent determinations ± the standard error.

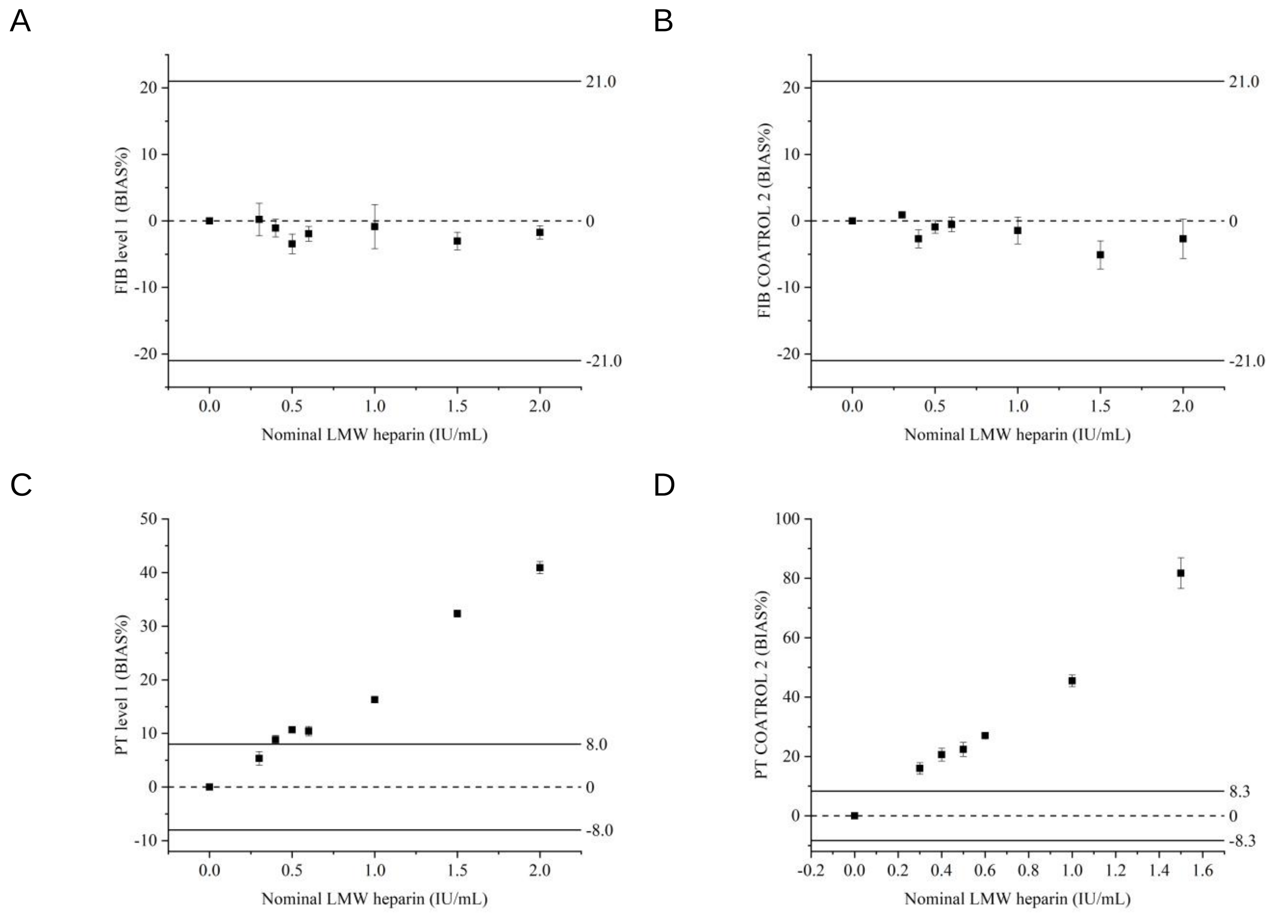

Supplement: Supplementary file 1 [file diagnostics-13-00085-s001.zip › diagnostics-2064952-SI.pdf]
